# Distribution of Phlebotomine Sandflies in the Cave Area of Satun Province, Thailand

**DOI:** 10.3390/tropicalmed5040174

**Published:** 2020-11-20

**Authors:** Suwich Thammapalo, Aulia Rahmi Pawestri, Kamal Kolaeh, Patcharida Boondej, Rittiporn Benarlee, Chamnarn Apiwathnasorn, Rawadee Kumlert

**Affiliations:** 1 The Office of Disease Prevention and Control 12, Department of Disease Control, Ministry of Public Health, Songkhla 9000, Thailand; sthammapalo@yahoo.com (S.T.); mang.kingdom@gmail.com (K.K.); banana0007@hotmail.com (R.B.); 2 Department of Disease Control, Ministry of Public Health, Nonthaburi 11000, Thailand; patcharida.gibb@gmail.com; 3 Faculty of Tropical Medicine, Mahidol University, Bangkok 10400, Thailand; aulia_rp@ub.ac.id (A.R.P.); chamnarn.api@mahidol.edu (C.A.); 4 Department of Parasitology, Faculty of Medicine, Universitas Brawijaya, Malang 65145, Indonesia

**Keywords:** leishmaniasis, sandfly species distribution, vector abundance, potential breeding site, environmental parameters, vector surveillance

## Abstract

Leishmaniasis, a sandfly-transmitted protozoan infection, is a neglected health threat in Thailand and the information on its vector is scarce. This study aimed to identify sandfly distribution, abundance, and environmental conditions of natural breeding sites in the cave areas of Satun Province, where previous cases of leishmaniasis were reported. Sandflies were collected during a six-month period using CDC light traps and modified emergence traps. Species distribution, relative abundance, and environmental conditions of potential breeding sites were determined. Our survey of 12,790 sandflies found the highest female abundance in April–May. We identified six known species, the most prevalent being *Sergentomyia anodontis*. We also found *S. barraudi*, a potential *Leishmania* spp. vector, distributing in this area. Most male sandflies had partially rotated genitalia, indicating the breeding site proximity to our trap locations. Potential resting/breeding sites were discovered outside the cave during February–March, and inside during May–June. The environmental parameters showed warm climate, moderate humidity, moderately alkaline pH, moderate-to-high macronutrients, and low-to-high organic matters. In summary, our study provided the spatiotemporal distribution and environmental condition of sandfly potential breeding sites in the cave areas of Satun Province. This data may contribute to more effective vector surveillance programs in the future.

## 1. Introduction

Leishmaniasis is a neglected public health threat in Thailand. The causative agent of this disease is *Leishmania* spp., an intracellular protozoon of the Family Trypanosomatidae. Until 1999, leishmaniasis was reported sporadically, until it was recognized as autochthonous infection, with most cases present in the southern part of Thailand [1,2]. Although leishmaniasis cases had been reported in Thailand since 1960 [3,4,5], this disease did not receive much attention due to its low prevalence.

*Leishmania* spp. is transmitted by female Phlebotominae sandflies. Up to 2016, 26 sandfly species had been reported in Thailand [6]. Recently, we performed an extensive literature search by including available data from 1934 to 2019 and discovered that at least 34 sandfly species are circulating in Thailand [7]. Two species, *Sergentomyia gemmea* and *S. barraudi*, were reported as potential vectors in Thailand [2,8], while *Phlebotomus argentipes* was identified as a confirmed vector of *Leishmania donovani* [9].

In South-East Asia, sandflies usually inhabit areas with high humidity and shades, such as caves, shrubs, wood piles, or forests [10,11,12]. In these areas, new species and high densities of sandflies could usually be found. Since appropriate environmental conditions support leishmaniasis transmission, the information on sandfly natural habitat and distribution is crucial. Additionally, surveys of natural potential breeding sites are necessary to explore the ecological markers for sandfly development. This information could contribute to vector survey planning and disease reservoir exploration during a disease investigation. Unfortunately, not many published studies addressed these issues in Thailand [4].

Satun Province, located in the southern part of Thailand, is a popular tourist destination. Leishmaniasis cases in the young population (1–15 years), which belonged to the known risk group, had been documented in this province and *L. martiniquensis* was shown to be the causative species [4]. Several species of sandflies had also been reported in this province [13,14]. Based on the above information, this research aimed to identify species distribution and relative abundance of sandflies in this area. Furthermore, we also explored the environmental condition of potential sandfly breeding sites. The data on sandfly distribution, abundance, and habitat could lead to more effective surveillance plans in the future.

## 2. Materials and Methods

### 2.1. Study Site and Environmental Parameter Assessment

**Figure 1:**
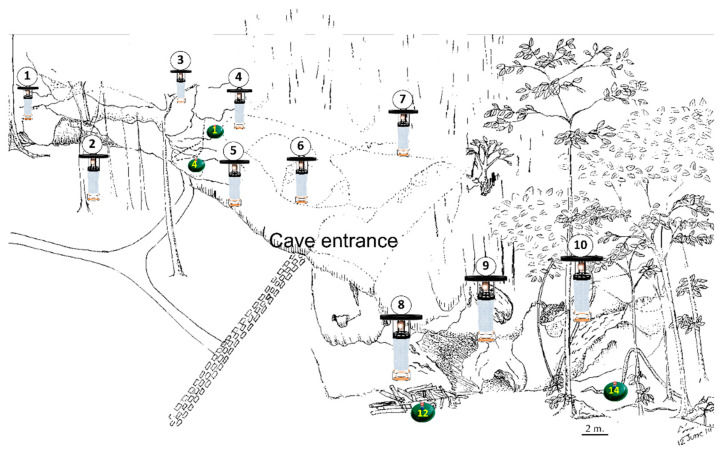
Trap positions in the study site. Ten CDC light traps (white) were positioned around the area of Tham Rakhang Thong Cave, Satun Province, Thailand. Additionally, emergence traps were also located inside (ten traps) and outside (eight traps) the cave. Four emergence traps with positive findings are shown in green.

Satun Province is located at the Malay Peninsula, on the shore of the Andaman Sea. The province is surrounded by sea, mountains, forests, caves, waterfalls, and wildlife sanctuaries [15]. There is significant rainfall on most months throughout the year, with only a short period of dry season. The average annual temperature is 27.3 °C, with annual rainfall of 2266 mm (89.2 inch) [16]. This area is famous not only for its natural attractions, but also due to the rock caves that serve as religious tourism spots. Based on previous surveillance reports, this area reported leishmaniasis cases [4] and high number of sandflies [13,14]. The study area was specifically the limestone cave areas of Tham Rakhang Thong Cave (7.094814, 99.917583) since caves were reported to harbor high numbers of sandflies and provide a suitable breeding site [9]. Traps were set inside and outside the cave, in locations with high humidity and scattered organic matter (Figure 1). The data was collected for six months, during February to July 2019, following a previous survey in which this period showed the high abundance of sandflies [14].

Temperature (T) and humidity (H) were recorded in 18 spots inside and outside the cave. The measurements were performed twice (morning and afternoon) at the same day of sandfly collection by digital thermo-hygrometer (Hig-MY TA290, WolfGo, Hubei, China). Soil samples were collected from the sites near the emergence traps once in the fifth month to evaluate the soil quality, including pH, organic matter (OM), nitrogen (N), phosphorus (P), and potassium (K). The soil quality assessment was kindly performed by the Land Development Regional Office 12, Thailand.

This study was approved by the institutional review board of Department of Disease Control, Thailand (ethical approval number 2/56-604/Version 1.2 dated 27 November 2012 and 62030 version 2 dated 22 March 2019).

### 2.2. Sandfly Abundance

Ten CDC light traps were settled in the study site once a month from 16.00 to 06.00 (Figure 1). Small insects and organisms captured in the CDC light traps were knocked down using chloroform. Sandflies were separated from other insects under the stereo microscope and counted. Male and female sandflies were separated and preserved in 70% ethanol. Approximately 50 of male and 100 of female sandflies per month were randomly sampled for permanent slide preparation. Specifically, 5 to 10 male and ten female sandflies per trap were collected from each designed CDC light trap. The male sandflies were used for observation of external genitalia rotation, while female sandflies were used for species identification.

### 2.3. Potential Breeding Site Exploration

**Figure 2:**
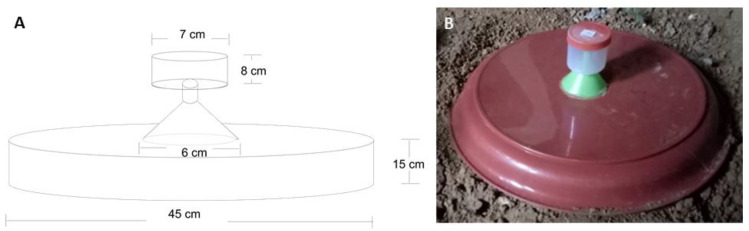
Modified emergence trap to determine potential breeding sites. (**A**) The schematic design of the modified emergence trap consisted of a tray, a plastic funnel, and a closed transparent container on top. (**B**) The modified emergence trap used in this study. They were placed to cover suspected breeding sites.

Potential breeding sites were explored using modified emergence traps [17]. The traps were positioned in 18 locations, including ten inside the cave and eight outside the cave (around the cave shelters and under the tree shades), with a distance of about 10–15 m from each other, covering suitable areas for breeding sites. Among the ten traps inside the cave, two of them were placed near the entrance in the photic area of the cave, while the remaining eight were placed in the aphotic area of the cave (15–20 m from the cave entrance). Each modified emergence trap was prepared using a tray 15 × 45 cm in dimension, with a plastic funnel 6 cm in diameter placed on top. The narrow end of the funnel ends in a closed transparent cylindrical container with the size of 7 × 8 cm in dimension (Figure 2). Adult sandflies were expected to emerge at the transparent container on top of the funnel.

The modified emergence traps were set for at least two days per month and observed each day. Small insects or organisms emerging in the container on top of the funnel were collected. These organisms were knocked down. Sandflies were separated from other insects and preserved in 70% ethanol for permanent slide preparation and morphological identification.

### 2.4. Morphological Identification

Approximately 100 of non-engorged female and 50–100 of male sandflies from the CDC light traps per month were randomly sampled for permanent slide preparation. The head, wing, and lower part of abdomen were maintained in Hoyer’s medium for morphological identification using higher magnifications (400× and 1000×) in the compound microscope. Male sandflies were observed for their external genitalia rotation and classified as unrotated or partially rotated and fully rotated (180°) genitalia. Unrotated or partially rotated genitalia indicated young or juvenile male sandflies. The proportion of young males was calculated to estimate the possible presence of breeding sites around the light trap positions. Male sandflies from the emergence traps were also observed for their external genitalia to confirm the potential breeding site.

The permanent slide for morphological identification was prepared using Hoyer’s medium. Briefly, one drop of Hoyer’s medium was placed onto the object glass. Then, the dissected sandfly organs were set on the Hoyer’s medium. The heads were inverted to the ventral side, the wings were expanded, and the abdomen parts were set into the lateral side. Cover slips were placed above the samples. The slides were labeled with sample number, site, and date of collection. The permanent slides were left to dry for seven days at room temperature.

Since only female sandflies feed on blood and could act as vectors for leishmaniasis, only female sandflies were used for sandfly species identification. Three identification keys were used, from Galati (2017), Rispail and Leger (1998), and Lewis (1978). However, the key from Lewis (1978), “The phlebotomine sandflies (Diptera: Psychodidae) of the oriental region”, was used as the main reference [18,19,20]. Morphological identification is still preferred in this study since there are not many sandfly DNA sequences from Thailand in the public database that could be used as reference for species identification [6]. Moreover, morphological identification is still used as the reference technique for sandfly species identification although it is time consuming, laborious, and requires entomological expertise [21].

### 2.5. Leishmania spp. Detection

The monthly collected female sandflies were subjected to *Leishmania* spp. detection. The upper second and third segment of the abdomen, which were left from the permanent slide preparation, were preserved individually in 95% ethanol upon dissection. After the sandfly species was identified, the samples were pooled based on species in each month of collection. Phenol-chloroform DNA extraction was performed individually for each pool as described previously [22]. The extracted DNA was then subjected to polymerase chain reaction-restricted fragment length polymorphism (PCR-RFLP) of the 70 kDa heat shock protein (HSP70) as previously described. The PCR products were then digested using *HaeIII*, separated in 2% agarose gel, and visualized using ethidium bromide staining [22,23,24].

### 2.6. Data Analysis

The data was presented descriptively. The trap success was calculated based on the average number of sandflies collected in each trap per night. The sandfly female abundance was determined by the number of female sandflies in each trap per night. The proportion of young or juvenile male sandflies was calculated by percentage of the unrotated or partially rotated male genitalia compared with the total male sandflies. The difference between soil parameters of positive and negative emergence traps and soil collected from inside and outside the cave was compared using the independent *t*-test, significance was set at *p* < 0.05.

## 3. Results

### 3.1. Sandfly Distribution and Abundance

**Figure 3:**
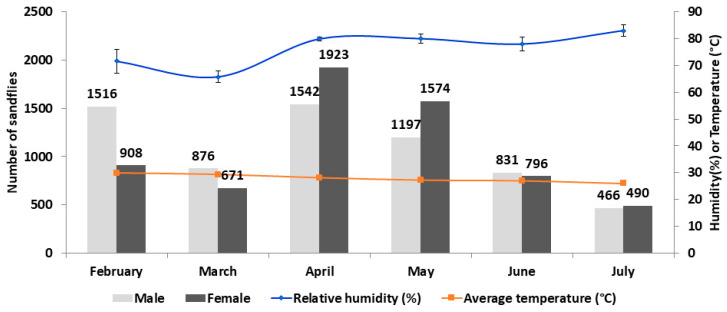
Monthly number of collected sandflies. The monthly number of male (grey bars) and female (black bars) sandflies is presented with the relative humidity (blue line) and average temperature (red line).

During the survey period of six months (February to July 2019), we collected 12,790 sandflies, which included 6428 (50.26%) males and 6362 (49.74%) females. Out of the total female sandflies, 369 (5.8%) of them were blood engorged. Monthly sex ratio showed males to be more prevalent than females in February to March (1.7–1.3:1). In April to May, females were more prevalent (0.8:1), while equal male to female ratio was found during June to July. The average trap success was 213 sandflies/trap/night and the average female abundance was 106 sandflies/trap/night. The highest trap success (347 sandflies/trap/night) and female abundance (192 sandflies/trap/night) was found in April. During the collection, the relative humidity was 80%, with an average temperature of 28 °C (Figure 3 and Table 1).

We randomly selected 598 non-engorged female sandflies for morphological identification. Using morphological identification keys, we identified six known sandfly species and several unknown species under two genera, *Sergentomyia* spp. and *Phlebotomus* spp. (Table 2). The species with the highest relative abindance was *S. anodontis* (26.8%). We also found *S. barraudi* (6.4%), which was previously reported as a potential *Leishmania* spp. vector in Thailand [2,8]. Other species included *P. asperulus* (5.9%), *P. stantoni* (1.8%), *S. silvatica* (0.8%), and *P. betisi* (0.2%). *S. anodontis* and *P. asperulus* could be found every month, while other species seemed to be restricted in specific months. Although we found a species of potential vector, we did not detect *Leishmania* spp. in any sample pool using the PCR-RFLP method.

### 3.2. Potential Breeding Sites and Environmental Parameters

To determine the possibility of the presence of potential breeding sites around the trap setting area, we randomly selected 545 male samples for external genital rotation observation. In each month, we found most male sandflies having unrotated or partially rotated genitalia (71–86%), showing that most of them were young or juvenile sandflies which had just emerged (Table 3). This indicated that the breeding places were close to our trap setting area since young or juvenile sandflies might have not flown very far from their breeding places.

**Figure 4:**
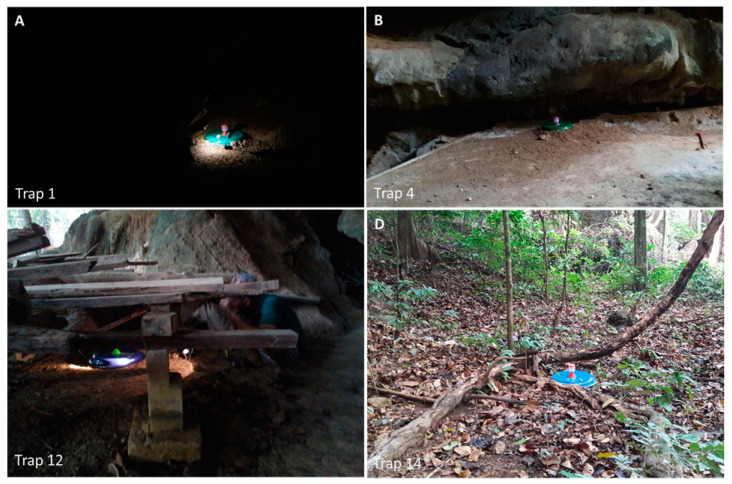
Sandfly resting or potential breeding sites around the cave area. Resting sites and potential breeding sites were found inside in the aphotic area of the cave (**A**), near the cave entrance (**B**), and outside the cave under the wood piles and tree shades (**C**,**D**), respectively.

Sandflies appearing from emergence traps were also collected. In February and March, when there was relatively lower humidity (68–77%) and higher temperature (28–30 °C), we found potential breeding sites outside the cave, such as under wood piles and tree shades. The potential breeding sites were identified by finding males with unrotated or partially rotated genitalia from the emergence traps. In May and June, with higher humidity of 80–82% and lower temperature (27 °C), sandflies were found in emergence traps inside the cave. We found one female sandfly in one out of two emergence traps near the cave entrance in the photic area of the cave. We found another female in one out of eight emergence traps in the aphotic part of the cave (15–20 m from the cave entrance), indicating these spots as resting sites or potential breeding sites (Table 4, Figure 4).

**Figure 5:**
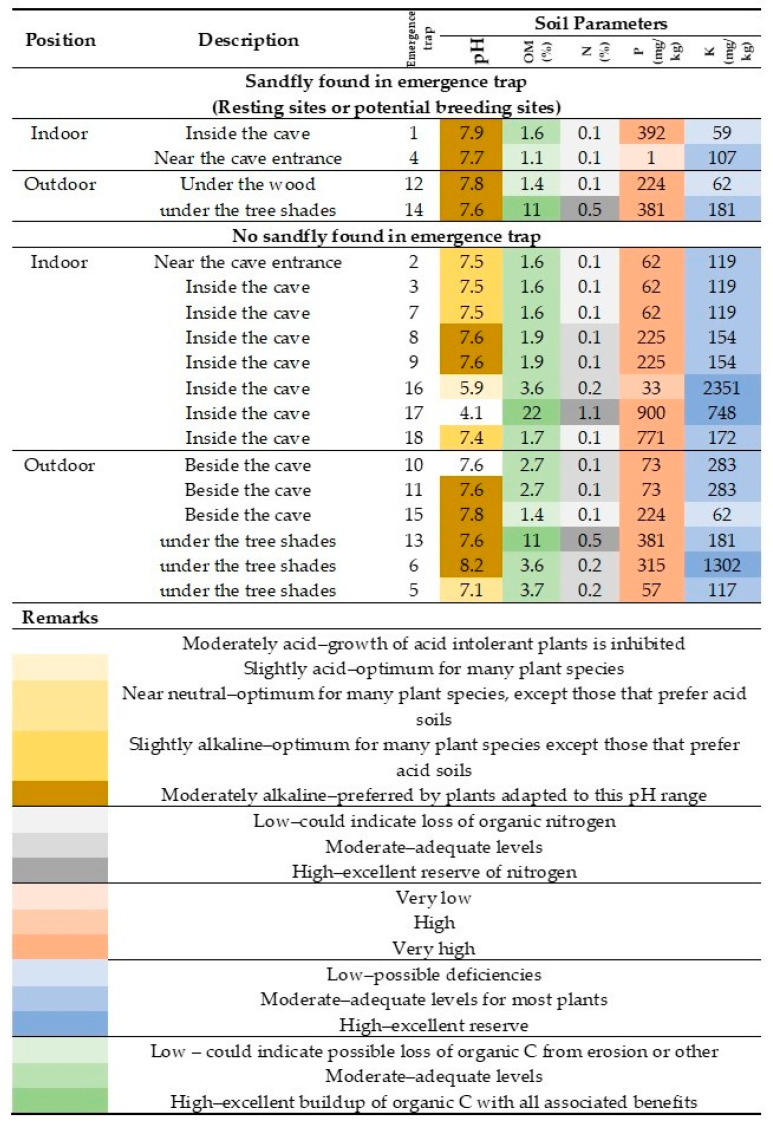
Soil parameters of potential sandfly breeding sites. Soil samples collected from emergence traps were assessed for levels of pH, organic matter (OM), nitrogen (N), phosphorus (P), and potassium (K).

The emergence trap is generally used to capture newly emerged adult sandflies from their potential breeding places in the soil [25]. The measurement of physical and chemical properties of the soil could provide information on the soil quality in sandfly breeding places. In this study, soil quality assessment in emergence traps where sandflies were found showed pH 7.6–7.9, organic matter 1.08–10.89%, nitrogen 0.05–0.54%, phosphorus 224–392 mg/kg, and potassium 59–181 mg/kg (Figure 5). This corresponds to moderately alkaline pH, moderate-to-high levels of macronutrients (N, P, and K), and low-to-high levels of organic matters. On the other hand, soil quality assessment in emergence traps where no sandflies were found showed a wider range of pH (4.1–8.2), organic matter 1.4–22%, nitrogen 0.1–1.1%, phosphorus 33–900 mg/kg, and potassium 62–2351 mg/kg (Figure 5). We performed an independent *t*-test analysis to see the differences in soil parameters in positive and negative emergence traps. We found no differences in pH (*p* = 0.16), organic materials (*p* = 0.43), nitrogen (*p* = 0.44), and phosphorus (*p* = 0.5). However, there was a slightly significant difference in potassium levels (*p* = 0.04) between the two groups. We also compared soil parameters collected from inside and outside the cave. There were no differences for all parameters [pH (P = 0.09), organic materials (*p* = 0.38), nitrogen (*p* = 0.45), phosphorus (*p* = 0.31), and potassium (*p* = 0.37)].

## 4. Discussion

Through a six-month survey period, our study reported the sandfly species distribution and relative abundance, monthly female abundance, and environmental conditions of potential breeding sites in Satun Province, Thailand, an area with previous reports of leishmaniasis cases.

During February to July 2019, we found 12,790 sandflies. Thailand only recognizes occasional cases of leishmaniasis. Nevertheless, the high sandfly abundance indicates that vector surveillance studies are necessary to further explore the problems of leishmaniasis in Thailand. We found the high ratio of male to female in February to March, while female to male ratio were highest during April to May. These results were supported by a study conducted in Egypt which showed that non-biting male sandflies were predominant during the early of dry season, whereas biting female sandflies become more abundant in the late period of dry season [26].

We identified six known sandfly species and other unidentified species belonging to two genera, *Sergentomyia* spp. and *Phlebotomus* spp. The highest relative species abundance was *S. anodontis* (26.8%). Other known species included *P. asperulus*, *P. betisi*, *P. stantoni*, *S. barraudi*, and *S. silvatica*. Our result was different from a study by Panthawong in 2015, which found *S. gemmea* (57.2%) to be the most frequent species in Satun Province, followed by *S. indica* (26.9%), *S. barraudi*, *S. stantoni*, and *S. iyengari*, respectively [14]. The list of the recently updated sandfly species in Thailand was presented in 2016. Covering a period of 1934 until 2012, it displays 26 species found in Thailand [6]. We performed an extensive literature review from 1934 until 2019 and updated the list into 34 sandfly species in Thailand [7].

Ninety-eight out of approximately 800 discovered sandfly species had been reported as confirmed or suspected vectors of leishmaniasis [27]. In Thailand, only two species had been reported to be the potential vector of leishmaniasis based on the identification of live parasites in the vector: *S. gemmea* and *S. barraudi* [2,8]. In this study, we found *S. barraudi* accounting for 6.4% of all identified species in the cave areas of Satun Province. The cave of our study site was located in a monastery area that was often used as a meditation place by the monks and visitors. Beside the dogs that were fostered by the monastery, bats were also commonly found inside the cave. These mammals could become potential reservoir hosts for *Leishmania* transmission, since a previous study described that certain sandfly species feed on a wide range mammalian blood, such as *S. barraudi* that was found to feed on elephants and humans [28]. Although in this study we did not detect *Leishmania* spp. in any of the sample pool due to the relatively low number of samples, the distribution of this potential vector might explain the transmission and reported cases of leishmaniasis in this province.

Regarding the monthly distribution, we identified two species that were observed every month: *P. asperulus* and *S. anodontis*. Other species seemed to be restricted in specific months only. A previous study on the distribution of sandflies in limestone caves also found *S. anodontis* to be present every month during October to September [9]. In 2011, a study on cave-dwelling sandflies also found this species during January to April in Phitsanulok Province, Thailand [29]. Moreover, during August 2005 to July 2006, the distribution of female sandfly species in caves in Saraburi Province showed *S. anodontis, S. barraudi, S. iyengari*, and *S. gemmea* to be present every month [30].

The highest trap success and female sandfly abundance was found in April (347 and 192 flies/trap/night, respectively). This was in accordance with a study by Polseela (2011) about cave-dwelling sandflies in Phitsanulok, Thailand, which reported the highest sandfly peaks during March and April [29]. Another study mentioned that sandflies were most abundant in the late dry season and early rainy season (April to June) [14].

In this study, we found that most male sandflies had unrotated and partially rotated genitalia. These juvenile males could give a hint on the location of natural sandfly breeding sites during vector surveillance. A previous study discovered that the duration of genitalia rotation differs among species, which ranged from 12 h in *S. schwetzi* to 33 h in *P. sergenti*. Moreover, the duration of rotation was influenced by the ambient temperature, in which lower temperature was shown to delay the initiation of rotation. Moreover, the behavior of the newly emerged males also differed among species. *Sergentomyia* spp. was immediately active, while *P. orientalis* stayed calm until they reached the mature stage [31]. This might explain the dispersal and abundance of the young males of certain species captured in emergence or CDC traps.

The hopping behavior estimates that sandflies do not disperse far from the breeding site [32]. Only host seeking or unfed females typically travel a few kilometers from their breeding site, while others rarely move more than a few hundred meters [33]. Our result showed that in the cave areas in Satun Province had potential sandfly breeding habitats. High proportion of males with non-inverted or partially inverted genitalia by CDC light traps suggested that our lights traps were placed near breeding sites.

The temperature and humidity ranges at the cave areas of Satun Province during February to July were 26–30 °C and 66–83%, respectively. For most sandfly species, the optimum temperature is between 24–28 °C, with a high humidity of 70–95%. However, these data were obtained from controlled conditions for sandfly colonization in the laboratories [10]. Thus, natural variation and differences among species also needs to be taken into consideration.

During April with the highest number of sandflies collection, the average temperature was 28 °C. Climate factors, such as temperature, affects an insect’s development, metabolic rates, egg production, the survival of pre-imaginal stages, and adult’s longevity and frequency of blood-feeding [34,35]. The amount of accumulated degree days (ADD) was found to be related to populations of sandflies. A study in Iran found that sandflies complete their life cycle and growth of the next generation in a total of 639 ADD. The first adult population appeared in the mid to late June of the next year when they receive at least 182 ADD from the beginning of the spring. The highest abundance of sandflies was observed in early August (21–24 °C), followed by a rapid decrease in early September (average temperatures 26 °C), and disappeared completely in late September. These results indicated that the environment temperature and ADD was closely related to sandfly population dynamics [36].

The information on environmental conditions of sandfly breeding site is important for strategic planning and implementation of vector surveillance programs [37]. In this study, we identified potential breeding sites, indicated by young male sandflies, in two locations outside the cave during February and March, which was located under the wood piles and tree shade. In May and June, we found two female sandflies in the emergence traps, indicating resting or potential breeding site inside the cave. A study in Sri Lanka reported sandfly resting sites in bushes, termite mounds, cattle huts, piles of coconut shells, latrines, and tree holes. Predominant breeding places were identified in mud flats and moist soils of rice paddies, the soil below decaying hay, drying irrigational tank bottom, and the floor of cattle huts [37]. These results was related to another study, which identified sandfly breeding site in five microhabitats (tree bases, unsheltered forest floor, soil from under fallen logs, soil from under roots, and palm-tree bases) [11].

In the forest environment, most sandfly breeding sites are located in highly humid microhabitats, such as tree bases, unsheltered forest floor, soil under fallen logs, soil under roots, and palm-tree bases [11]. In our study, the sites supposedly to be potential sandfly breeding sites had a moderately high humidity of 68–82% and temperatures of 27–30 °C, which might provide a suitable breeding place for the phlebotomine larvae.

The soil quality had moderately alkaline pH, moderate-to-high macronutrients, and low-to-high organic matters. Slightly to moderately alkaline soil pH has been shown to provide optimal growth of the microenvironment. Thus, this condition might provide a supply of micronutrients for the nourishments of the sandfly larvae, thus promoting their development [38]. Aside from the potassium levels, there were no significant differences between soil parameters taken from sites near the positive and negative traps. There were also no differences in soil quality taken from inside or outside the cave.

## 5. Conclusions

Taken together, our study provided the spatiotemporal distribution, abundance, and potential breeding sites of sandfly species in the cave area of Satun Province. We provided the environmental data related to the potential breeding sites, which were spread around and possibly inside the caves. This data could contribute to formulation of strategic plans for vector surveillance programs to enrich the sandfly distribution data for further studies in the future.

## Figures and Tables

**Table 1:** Male-to-female ratio, trap success, and female abundance.

Month	Relative Humidity (%) (Mean ± SD)	Average Temperature (°C) (Mean ± SD)	Total Number (Male:Female)	Blood Engorged Female	Trap Success ^1^	Female Abundance ^2^
February	72 ± 4.51	30 ± 0.77	2424 (1.7:1)	12 (1.3%)	242	91
March	66 ± 2.16	29 ± 1.05	1547 (1.3:1)	45 (6.7%)	155	67
April	80 ± 0.70	28 ± 0.64	3465 (0.8:1)	118 (6.2%)	347	192
May	80 ± 1.61	27 ± 0.58	2771 (0.8:1)	128 (8.2%)	277	157
June	78 ± 2.48	27 ± 0.55	1627 (1:1)	49 (6.1%)	163	80
July	83 ± 2.09	26 ± 0.63	956 (1:1)	17 (3.5%)	96	49
Total			12790 (1:1)	369 (5.8%)		

^1^ Trap success: average number of sandflies/trap/night; ^2^ Female abundance: average number of female sandflies/trap/night.

**Table 2:** Monthly distribution and relative abundance of sandfly species.

Species	February	March	April	May	June	July	Relative Abundance (%)
*S. anodontis*	28	20	19	19	36	38	160 (26.8%)
*S. barraudi*	25	3	2	0	1	7	38 (6.4%)
*S. sylvatica*	0	0	0	4	0	1	5 (0.8%)
*Sergentomyia* spp. ^1^	38	57	62	59	40	34	290 (48.5%)
*P. asperulus*	6	6	5	2	10	6	35 (5.9%)
*P. stantoni*	1	4	3	3	0	0	11 (1.8%)
*P. betisi*	1	0	0	0	0	0	1 (0.2%)
*Phlebotomus* spp. ^2^	12	6	7	10	11	12	58 (9.7%)
	111	96	98	97	98	98	598

^1^*Sergentomyia* spp. (possible to be seven species); ^2^ *Phlebotomus* spp. (possible to be two species), still waiting for confirmation.

**Table 3:** Proportion of external genitalia rotations of male sandflies.

Month	Total Number	Numbers of Samples Selected	Unrotated or Partially Rotated Genitalia	Fully-Rotated Genitalia
February	1516	53	43 (81%)	10 (19%)
March	876	71	52 (73%)	19 (27%)
April	1542	95	80 (84%)	15 (16%)
May	1197	113	80 (71%)	33 (29%)
June	831	111	96 (86%)	15 (14%)
July	466	102	78 (76%)	24 (24%)
Total	6828	545	429 (78.7%)	116 (21.3%)

**Table 4:** Locations of sandfly resting sites or potential breeding sites.

Trap	Location of Resting Sites or Potential Breeding Sites	Month	Presence of Sandfly
Number			
01	Inside the cave (aphotic area)	June	1 female *S. anodontis*
04	Near the cave entrance (photic area)	May	1 female *Sergentomyia* sp.
12	Outside the cave, under the wood piles	February	1 male partially rotated genitalia
14	Outside the cave, under the tree shades	March	1 male partially rotated genitalia
